# Extensive Cutaneous Scalp Angiosarcoma

**DOI:** 10.1155/2018/8409820

**Published:** 2018-06-21

**Authors:** Zabeer Bhatti, Rameez Bhatti, Sharon Brangman, Kerry Whiting, Amit Dhamoon

**Affiliations:** ^1^State University of New York Upstate Medical University, 750 E. Adams Street Syracuse, NY 13210, USA; ^2^Avalon University School of Medicine, 122-124 Santa Rosaweg, Curacao, Netherlands

## Abstract

Angiosarcoma is a cancer that is derived from endothelial cells that line blood vessels and lymphatic channels. Cutaneous angiosarcoma can appear anywhere on the skin and the clinical presentation is highly variable. Most cases appear on the scalp and face de novo. Our case describes a 91-year-old female with cutaneous scalp angiosarcoma. Our case serves to remind physicians that an abnormal skin finding in older adults should raise their index of suspicion for angiosarcoma and an early biopsy should be performed.

## 1. Introduction

Angiosarcoma is a highly invasive tumor with a poor prognosis. Ten-year survival rates of scalp angiosarcoma have been reported to be as low as 13.8% with metastatic disease and as high as 53.6%, if the disease is localized [1]. Early detection is therefore essential to the treatment. The following case is reported because of the several clinical diagnoses that were mistakenly given prior to the correct diagnosis of angiosarcoma provided. Given the aggressive nature of this tumor, a delay in the definitive diagnosis may have played a role in the malignant course and therefore treatment options.

## 2. Case Presentation

**Figure 1:**
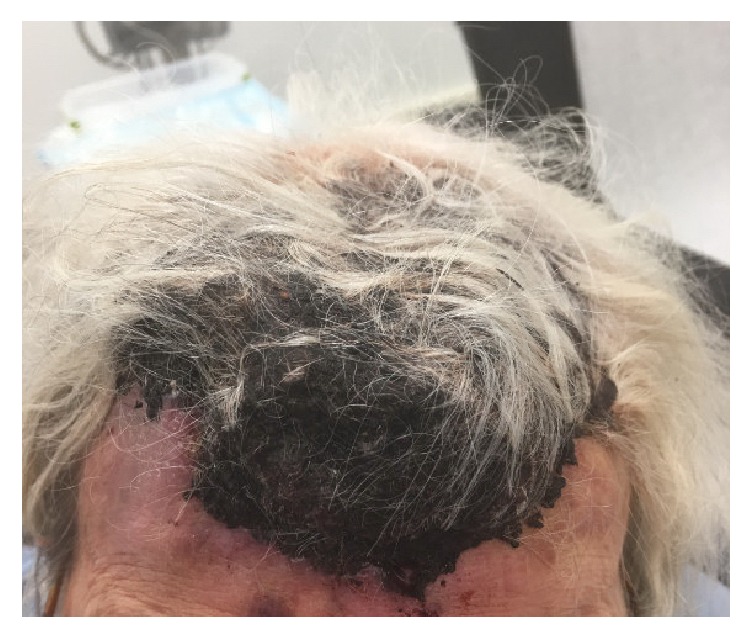
A bluish-violaceous discoloration is seen under her scalp.

**Figure 2:**
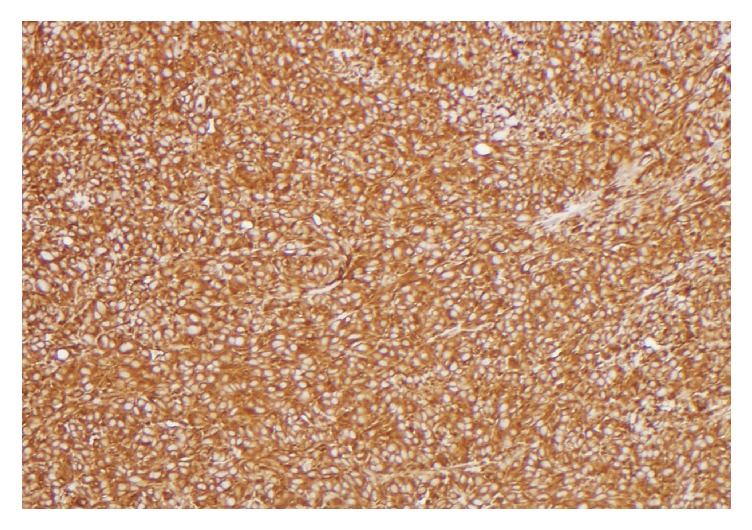
The cells are uniformly positive for vimentin, a nonspecific stain that confirms the mesenchymal origin of tumors.

**Figure 3:**
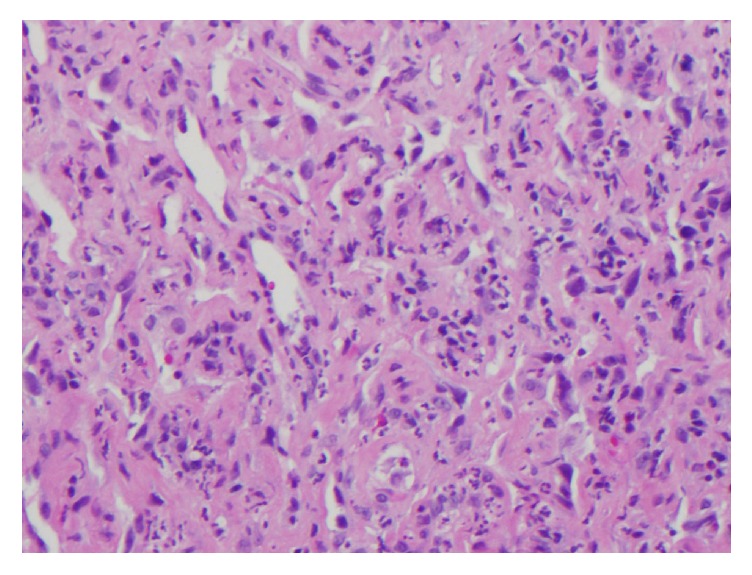
The anastomosing channels are readily apparent and are lined with atypical endothelial cells with enlarged and hyperchromatic nuclei.

**Figure 4:**
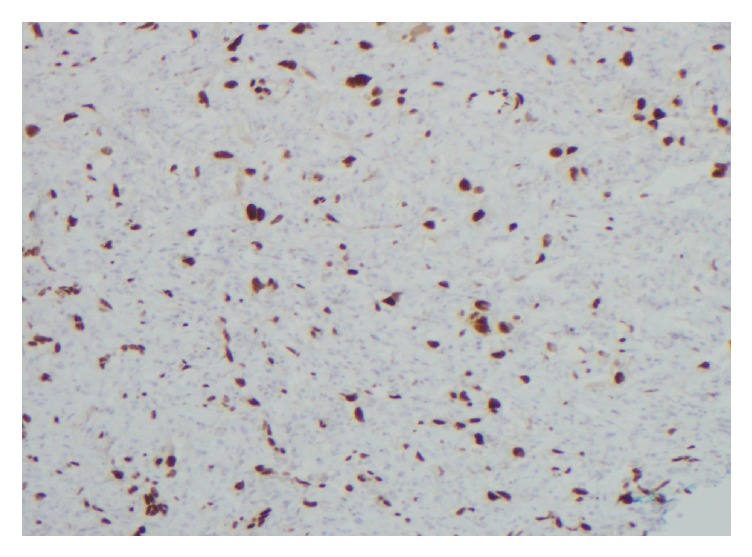
The neoplastic cells also express CD31, a relatively sensitive and specific marker for endothelial cells that is also positive in vascular tumors, including angiosarcoma.

**Figure 5:**
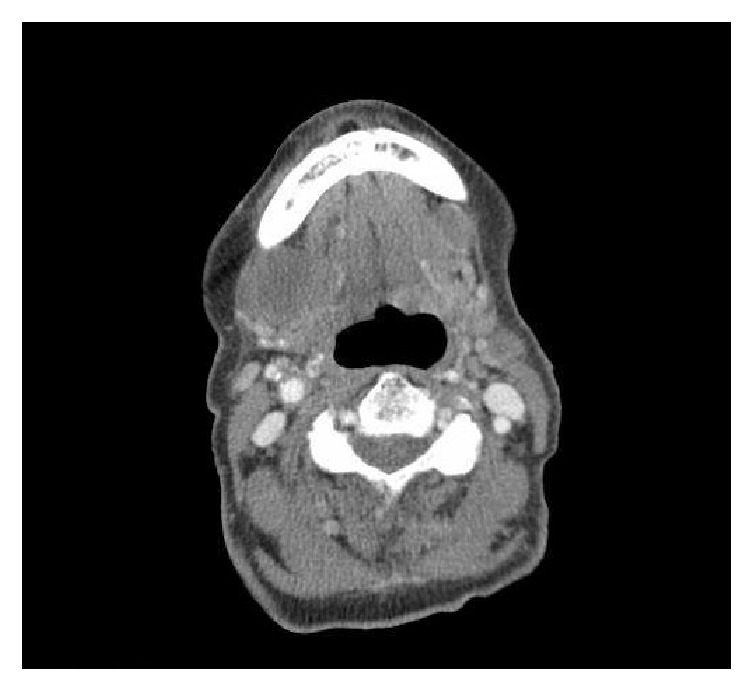
A 2.8 cm focal lesion was identified on the anterior aspect of the right submandibular gland representing an enlarged lymph node. A smaller, 1.6 cm enlarged lymph node was also present on the left side.

A 91-year-old female was admitted to the hospital after suffering a burn to her forehead by a salon hair dryer approximately four months prior to diagnosis. Following the development of the burn, she reported pruritic red patches appearing on the scalp. Over the four months, several clinical diagnoses, including eczema, cellulitis, and hematoma, were rendered by her primary care physician. For her presumed diagnoses, courses of therapy including topical hydrocortisone, moisturizers, and antibiotics were prescribed. While she was compliant with the outlined regimen, her symptoms did not improve. She was then referred to the hospital after she reported mild but persistent bleeding from the lesions. On initial examination, a large purplish mass on the skin of the frontal scalp was identified [Figure 1]. An incisional biopsy was performed, which revealed angiosarcoma [Figures 2, 3, and 4]. Computerized tomography was ordered for evaluation in case of metastatic disease. Imaging showed frontal scalp swelling with multiple enlarged lymph nodes concerning metastatic disease [Figure 5]. She was not interested in pursuing surgery or chemotherapy but agreed to consider radiation therapy to control the bleeding and be comfortable enough to wear a wig. We agreed with the treatment plan and offered palliative radiation for symptom control. The patient was treated with 4500 centigray (cGy) in 300 cGy daily fractions. She tolerated the treatment with no unanticipated side effects. After the intended course she had flattening of the tumor but with residual bleeding. She passed away at home a few weeks later.

## 3. Discussion

Wide local excision of the lesion for histological tumor-free margin is the treatment of choice for angiosarcoma [2]. Systemic chemotherapy followed by adjuvant radiation therapy is recommended in metastatic disease [2]. In our patient, however, given the extensive involvement of the tumor in the scalp and face and her age, complete resection was not feasible. She refused systemic chemotherapy but agreed to palliative radiotherapy to control the bleeding scalp lesions.

Cutaneous angiosarcoma has been described to be clinically variable in literature. Previously published case reports have described lesions as raised purplish-red papules, which can be misdiagnosed as rosacea, eczema, and hematoma [3–6]. Due to this variability in presentation, the correct diagnosis can be delayed, often drastically altering the outcome and treatment options. Prognosis is poor with a reported survival rate corresponding to the age, anatomical site, and stage of the disease [1].

In our case several clinical diagnoses, including burn, eczema, cellulitis, and hematoma, were mistakenly given prior to the correct diagnosis of angiosarcoma provided. A delay in the definitive diagnosis may have played a role in the malignant course and therefore limited her treatment options. Our case serves to remind physicians that an abnormal skin finding in older adults should raise their index of suspicion for angiosarcoma and an early biopsy should be performed.
